# Efficacy of probiotics in pediatric atopic dermatitis: A systematic review and meta‐analysis

**DOI:** 10.1002/clt2.12283

**Published:** 2023-07-15

**Authors:** Xiali Xue, Xinwei Yang, Xiubo Shi, Zhongyi Deng

**Affiliations:** ^1^ School of Sports Medicine and Health Chengdu Sport University Chengdu China; ^2^ Hospital of Chengdu Office of People's Government of Tibetan Autonomous Region Chengdu China; ^3^ Department of Rehabilitation Medicine The Third Affiliated Hospital Sun Yat‐Sen University Guangzhou China

**Keywords:** atopic dermatitis, children, meta‐analysis, probiotics, treatment

## Abstract

**Background:**

Atopic dermatitis (AD) is a prevailing skin disease in childhood. Several studies have appraised probiotics as a strategy for treating AD. We aimed to assess the validity of probiotics in the treatment of AD in children.

**Methods:**

We systematically searched the PubMed/MEDLINE, Embase, Scopus, EBSCO, Web of Science and Cochrane library databases for randomized controlled trials (RCTs) that assessed the effect of probiotic treatment on SCORAD value in pediatric patients with AD compared with a placebo group between 1 January 2010 and 1 January 2023. The risk of bias and the certainty of evidence were assessed using Cochrane ROB 2.0.

**Results:**

A total of 10 outcomes from 9 RCTs involving 1000 patients were included. Three of these outcomes were analyzed as dichotomous variables in 373 patients. The other seven were analyzed for continuous variables in 627 patients. A meta‐analysis of the random‐effect model of the dichotomous variables demonstrated no significant difference between the probiotic and control groups [OR = 1.75, 95% confidence interval (CI) (0.70, 4.35), *p* = 0.23, *I* ^2^ = 68%]. A meta‐analysis of the random‐effect model of continuous variables demonstrated significant differences between the probiotic and control groups [MD = −4.24, 95% CI (−7.78, −0.71), *p* = 0.002, *I* ^2^ = 71%]. Subgroup analysis of continuous variables showed that the effects of children's age, treatment duration and probiotic species on the SCORAD value were not statistically significant.

**Conclusion:**

Evidence on the improvement effect of probiotics on pediatric patients with AD is limited. This study showed that single‐strain probiotic treatment exerts a positive effect on AD. Restricted to the quantity and quality of incorporated studies, these conclusions have yet to be validated by high‐quality studies.

## 1 INTRODUCTION

Atopic dermatitis (AD) is a common inflammatory disease and the most prevalent chronic inflammatory skin disorder in children.^1^, ^2^ Its symptoms, including skin scratches and itching at night, considerably affect the quality of lives of children and their families.^3^ The incidence of AD has accelerated globally in the last decade, with approximately 10%–20% of children in developed countries living with the disease.^4^, ^5^ AD usually appears in early infancy and childhood. Diet, breastfeeding rates, and sanitation are critical factors in the progress of atopic diseases.^6^, ^7^ Microbiota dysbiosis is influenced by different environmental factors, including diet and Vitamin D (VD) exposure and intake. Maternal prenatal nutrition and dietary diversity are key factors in child development. The worldwide increase in allergic diseases parallels the prevalence of VD deficiency in Western countries, supporting the hypothesis that VD influences allergy development. VD levels are affected not only by sun exposure but also by diet, making it an important modifiable factor for allergy prevention.^8^ Similarly, the gut microbiota is thought to play a crucial part in the pathogenesis of atomicity.^9^, ^10^ Maintaining the structural diversity of the gut microbiota can resist the invasion of pathogenic bacteria and reduce the nutrient competition between potentially harmful bacteria and commensal bacteria. Gut microbiota involved in short‐chain fatty acid and amino acid metabolism induce maturation of the innate and adaptive immune systems. Thus, the gut microbiota is a potential target for modulating host immune responses. With the development of sequencing technology, many studies have revealed the correlation between gut microbiota and human diseases (including allergic asthma, atopic dermatitis). The “Gut‐Skin” axis has been proposed and considered as a new target for the prevention and treatment of AD.^11^ Gut microbiota is the most important component of microbial exposure. Therefore, gut microbiota affects the formation of host immune development, and gut microbiota dysbiosis is closely related to immune disorders. Therefore, targeting gut microbiome alterations may be an alternative approach to modulate the immune response and improve skin health in AD patients.^12^

In 2001, the World Health Organization defined probiotics as living microorganisms that have beneficial effects on the host when consumed in adequate amounts.^13^ Over the same period, Kalliomäki et al. conducted a randomized controlled study and found that perinatal consumption of the gram‐positive probiotic *Lactobacillus rhamnosus* (*Lactobacillus* GG) reduces the incidence of eczema in high‐risk infants by half. Thus, probiotics have gained attention for the treatment of AD. Later studies found that skin bacterial diversity decreases, *Malassezia* spp. and *Cutibacterium* (formerly *Propionibacterium*) acnes decrease and *Staphylococcus aureus* and *Staphylococcus epidermidis* are relatively abundant in children with AD.^14^ Gueniche et al. demonstrated that *Lactobacillus paracasei* CNCM I‐2116 (ST11) constricts skin inflammation and prompts the repair of the skin barrier.^15^ Kong et al. found through mouse experiments that *Corynebacterium mastitidis* and *Corynebacterium bovis* can improve the state of patients with AD, and relevant studies have proven that they can reverse the gut dysbiosis caused by the abuse of antibiotics in early treatment.^16^, ^17^ Probiotics may actively fight pathogenic bacteria by secreting antimicrobial factors. They also regulate the immune system and direct it to attack pathogenic microorganisms or promote immune tolerance, thereby reducing inflammation.^18^ Strengthening intestinal homeostasis may improve AD, and probiotics are increasingly being used in dermatology.

The application of probiotics to promote the scoring atopic dermatitis (SCORAD) value in children with AD remains controversial, and some studies have suggested that probiotics can effectively improve AD in children.^19^, ^20^ Another study found no significant difference between the experimental and placebo groups.^21^, ^22^ Therefore, the present work aimed to perform a systematic review and meta‐analysis of randomized controlled trials (RCTs) that used probiotics for AD treatment in children. The findings of this work provide evidence for probiotic effects on AD.

## 2 METHODS

This systematic review and meta‐analysis is a review of interventions conducted by the Cochrane System Manual and following the Preferred Reporting Item for Systematic Review and Meta‐Analysis (PRISMA) guidelines (see Supplementary Material 1).^23^ This review has been registered on the PROSPERO (CRD42023407446).

### 2.1 Eligibility criteria

All RCTs that evaluated the efficacy of probiotics in AD treatment were included based on the following criteria: (1) Participants aged ≤18 years, Meet the diagnostic criteria of the American Academy of Dermatology Diagnostic Criteria for Atopic Dermatitis,^24^ Chronic or relapsing history, Eczema (acute, subacute, chronic), Pruritus, Typical morphology and age‐specific patterns; (2) Probiotics taken orally with clear coverage of probiotic species, dose and timing of administration; (3) Control groups receiving placebo; (4) Primary endpoints at the time point of follow‐up after the intervention; (5) SCORAD value, a valid indicator for assessing the severity of AD; (6) This parameter should be included in the filtered experimental results for AD diagnostic criteria that meet the search criteria.

### 2.2 Exclusion criteria

(1) Non‐English literature; (2) Incomparability between the intervention and control groups; and (3) Letters, conference abstracts and comments.

### 2.3 Literature search strategy

The PubMed/MEDLINE, Embase, Scopus, EBSCO, Web of Science and Cochrane library were searched for RCTs (from 1 January 2010 to 1 January 2023). To search as comprehensively as possible, we applied a combination of free word and medical subject terms using the most appropriate Boolean operators to expand the scope of the study and find additional references. Keywords related to AD, probiotics, RCT and children in the title or abstract were searched. Using the PubMed database as an example, the following search terms are employed: Atopic Dermatitis (e.g., “Dermatitis atopic e” or “Dermatitides” or “Neurodermatitis atopic”); Probiotic (e.g., “Probiotics”); Randomized controlled trial (e.g., “Randomized” or “Randomly” or “Clinical trial”). The specific retrieval strategy is displayed in Supplementary Material 2. Two researchers (YXW and XXL) independently assessed all retrieved articles against eligibility criteria. In case of a disagreement, we will discuss it with the team until an agreement has been reached.

### 2.4 Data extraction

Data extraction was quality checked by two other study authors. Data from each study were extracted independently by two researchers (XXL and DZY), who then examined each other's results to avoid errors. The researchers used a standardized form of extraction of the article data, with an extraction table documenting the details of the study, including basic article information (author name, publication year, etc.), intervention characteristics (probiotic species, dosage) and placebo use (placebo type and measure).

### 2.5 Risk of bias assessment

The risk of bias was appraised for the primary outcome of AD using the Cochrane Risk of Bias tool using PRISMA guidelines. Assessments were mainly based on random sequence generation, whether the study used blinding, allocation concealment, completeness of data results, selective reporting of data, results and other sources of bias. The assessment was answered and explained through the form.

### 2.6 Statistical analysis

Data analysis was performed using Review Manager version 5.4.1. For measurement data, the standardized mean difference was used as the effective index, and point estimates and a 95% confidence interval (CI) were given for each effect size. Heterogeneity among the outcomes of the included studies was analyzed using the *χ* ^2^ test (the test level was *α* = 0.1), and the magnitude of heterogeneity was quantitatively judged by *I* ^2^. If the heterogeneity *I* ^2^ < 50%, the fixed‐effect model was used; if the heterogeneity *I* ^2^ > 50%, the random‐effect model was used. A descriptive statement of the study results was given when the synthetic analysis was not possible. After calculating the results, the combined results with significant heterogeneity were further subjected to sensitivity analysis, subgroup analysis and meta‐regression to help determine the source of heterogeneity. Publication bias testing was performed on outcome indicators with more than 10 included studies. Meta‐analysis of random‐effect or fixed‐effect models was used to estimate combined treatment effects. Subgroup analyses were based on children's age or treatment duration (e.g., ≤3 years or >3 years) and type of probiotics.

## 3 RESULTS

### 3.1 Study selection

**FIGURE 1:**
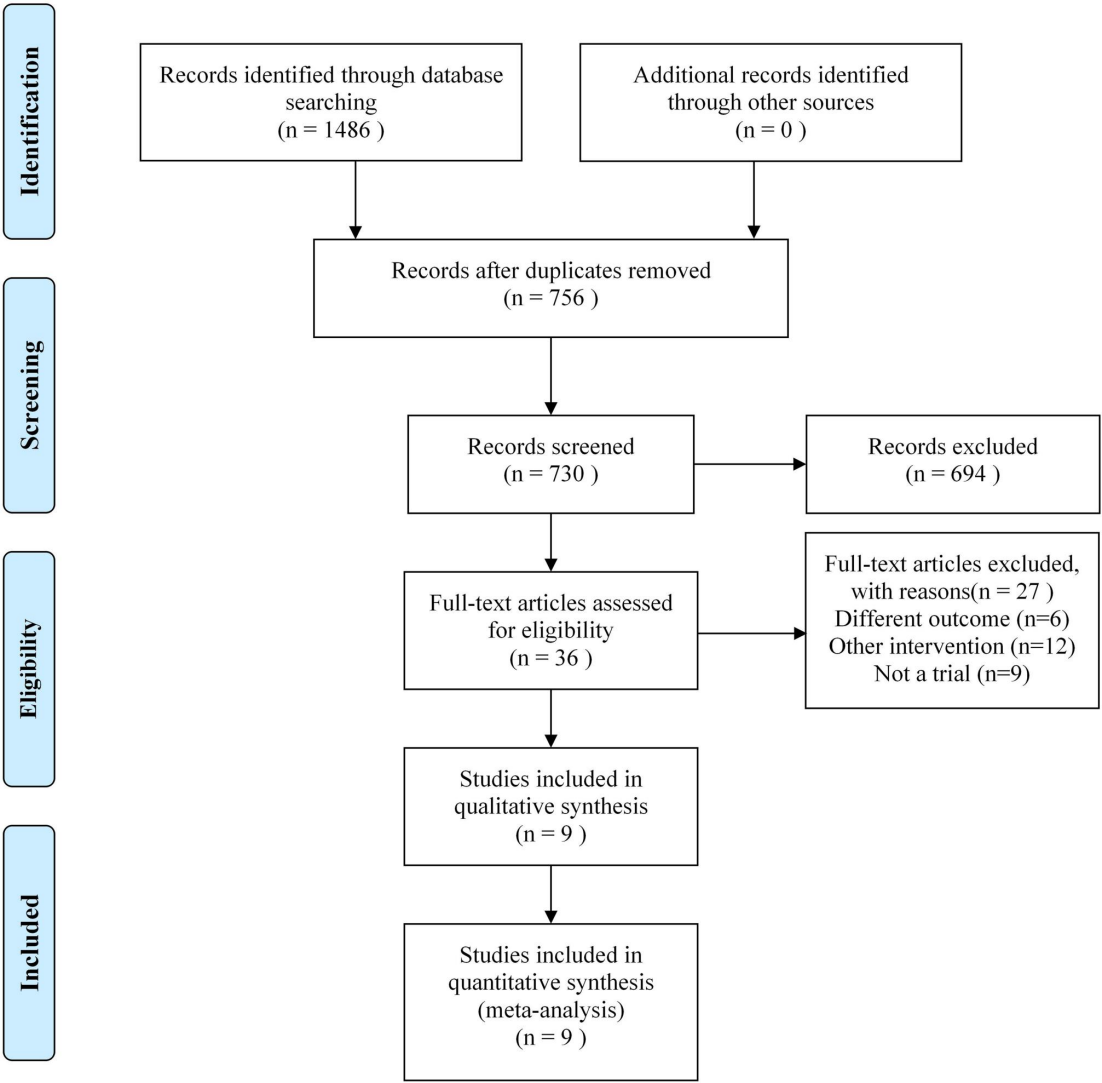
Flow diagram of the study selection process.

A total of 1486 studies were retrieved from the electronic database. Among these studies, 756 duplicate documents were removed, and 730 articles remained. We obtained the results of the first screening by excluding factors such as animal experiments, literature type review, inconsistent outcome indicators, duplication and incomplete data. A total of 53 cases met the conditions for full‐text review. After a careful review of the full text, 9 studies^25^, ^26^, ^27^, ^28^, ^29^, ^30^, ^31^, ^32^, ^33^ were finally included in the systematic review and meta‐analysis. The literature screening flowchart is shown in Figure 1. The main characteristics of the 9 included studies are shown in Table 1.

**TABLE 1:** Characteristics of the included studies in the meta‐analysis.

First author Year (Ref.)	Sample size (T/C)	Age, (T/C)	Intervention	Intervention duration	Outcomes
Gerasimov 2010	43/47	25.6 ± 7.7/24.1 ± 6.3 months	**T**: 1.0 × 10^10^ colony‐forming units of Lactobacillus acidophilus DDS‐1 and UABla‐12 twice daily	8 weeks	SCORAD
			**C**: 1.0 × 10^10^ colony‐forming units placebo twice daily		
Wu 2011	27/27	7.8 ± 3.5/6.9 ± 3.4 years	**T**: 475 mg fructo‐oligosaccharide plus 25 mg live probiotic (2 × 10^9^ colony‐forming units L. salivarius) twice daily	8 weeks	SCORAD
			**C**: 475 mg fructo‐oligosaccharide plus 25 mg corn starch twice daily		
Han 2012	58/60	4.6 ± 3.3/5.1 ± 3.3 years	**T**: 0.5 × 10^10^ colony‐forming units of L. plantarum CJLP133 twice daily	12 weeks	SCORAD
			**C:** 0.5 × 10^10^ colony‐forming units placebo twice daily		
Wang 2015	51/53	8.34 ± 3.80/8.04 ± 3.97 years	**T**: 4 × 10^9^ colony‐forming units of a mixture of Lactobacillus paracasei and Lactobacillus fermentum once daily	3 months	SCORAD
			**C**: 4 × 10^9^ colony‐forming units placebo once daily		
Navarro‐López 2017	26/24	9.35 ± 3.58/8.96 ± 3.94 years	**T**: 1.0 × 10^9^ colony‐forming units of a mixture of the 3 probiotic strains (Bifidobacterium lactis CECT 8145, B longum CECT 7347, and Lactobacillus casei CECT 9104) in a 1:1:1 ratio	12 weeks	SCORAD
			**C**: 1.0 × 10^9^ colony‐forming units placebo (maltodextrin) once daily		
Plummer 2019	118/137	Preterm infants (Birth to discharge from hospital or term CA)	**T**: 1.0 × 10^9^ colony‐forming units of a mixture of Bifidobacterium infantis, Streptococcus thermophilus, and Bifidobacterium lactis once daily	2 years	SCORAD
			**C**: placebo (maltodextrin) once daily		
Jeong 2020	33/33	5.67 ± 3.30/5.33 ± 2.53 years	**T**: 1.0 × 10^10^ colony‐forming units of Lactobacillus rhamnosus IDCC 3201 tyndallizate once daily	12 weeks	SCORAD
			**C**: 1.0 × 10^10^ colony‐forming units placebo once daily		
Ahn 2020	41/41	4.8 ± 2.3/5.4 ± 3.0 years	**T**: 1.0 × 10^10^ colony‐forming units of L. pentosus once daily	12 weeks	SCORAD
			**C:** 1.0 × 10^10^ colony‐forming units placebo once daily		
Cukrowska 2021	66/68	8.2 ± 6.1/8.8 ± 6.6 months	**T**: 1.0 × 10^9^ colony‐forming units of a mixture of three probiotic strains (Lactobacillus casei ŁOCK 0919, Lactobacillus rhamnosus ŁOCK 0908, Lactobacillus rhamnosus ŁOCK 0900)once daily	3 months	SCORAD
			**C**: 1.0 × 10^9^ colony‐forming units placebo (maltodextrin) once daily		

Abbreviations: C, Control group; T, trails group.

### 3.2 Study characteristics

Table 1 summarizes the essential characteristics of the included studies. Of the 9 RCTs from 2010 to 2021, 5 used multi‐strain mixtures to treat AD, and the other 4 used single‐strain interventions. The participants included in the study were children and infants aged 0–18 years, including a premature infant in one study. The most common intervention period in the included studies was 12 weeks. *Bifidobacterium lactis* is a commonly used probiotic strain, and the doses used for the probiotic strains in the trial population varied. However, 1.0 × 10^10^ colony‐forming units were the most frequently used dose in the included studies.

### 3.3 Risk of bias in the included studies

The risk of bias in the studies is presented in Table 2. The included studies were high‐quality RCTs, and most of the studies followed high standards: 5 (56%) appropriately generated random sequences, 3 (33%) applied appropriate approaches for allocation concealment, and 9 (100%) used blinding.

**TABLE 2:** The risk of bias of randomized controlled trials (RCTs) included and evaluated through Rob 2.0.

Author, year	Randomization process	Deviation from intended interventions	Missing outcome data	Measurement of the outcome	Selection of the reported result	Overall
Gerasimov 2010	Low risk	Low risk	Low risk	Some concerns	Some concerns	Some concerns
Wu 2011	Low risk	Low risk	Low risk	Low risk	Low risk	Low risk
Han 2012	Low risk	Low risk	Low risk	Some concerns	Low risk	Some concerns
Wang 2015	Low risk	Low risk	Low risk	Low risk	Low risk	Low risk
Navarro‐López 2017	Some concerns	Low risk	Low risk	Some concerns	Low risk	Some concerns
Plummer 2019	Low risk	Some concerns	Low risk	Low risk	Low risk	Some concerns
Jeong 2020	Some concerns	Some concerns	Low risk	Some concerns	Low risk	Some concerns
Ahn 2020	Some concerns	Low risk	Low risk	Some concerns	Low risk	Some concerns
Cukrowska 2021	Some concerns	Low risk	Low risk	Some concerns	Low risk	Some concerns

### 3.4 Clinical effect of probiotics

#### 3.4.1 Overall clinical effect

**FIGURE 2:**
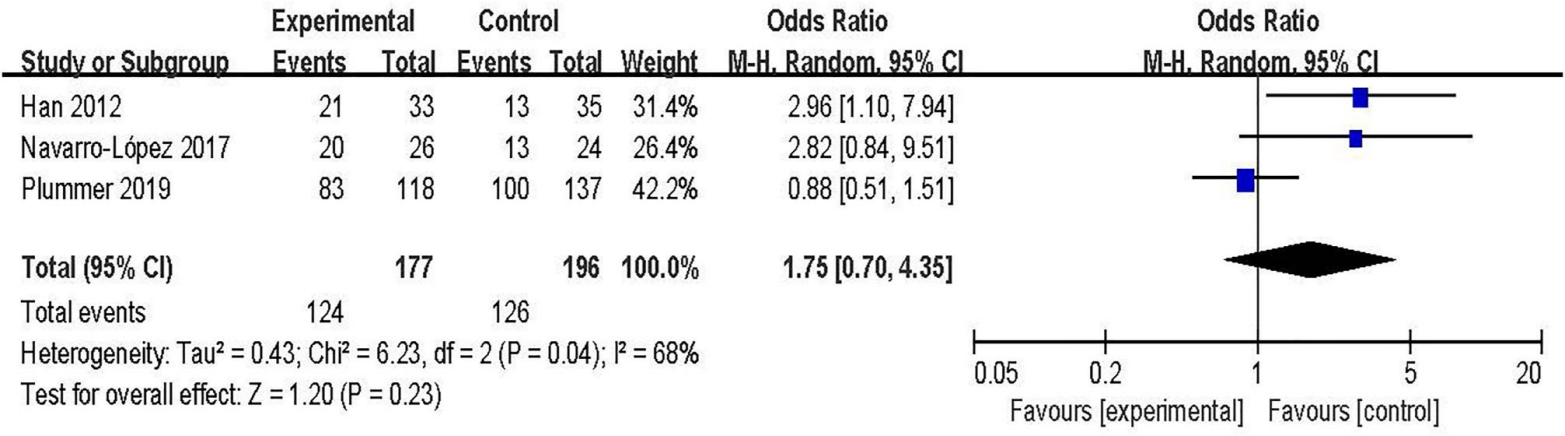
Forest plot showing the association between probiotics and Atopic dermatitis (AD).

**FIGURE 3:**
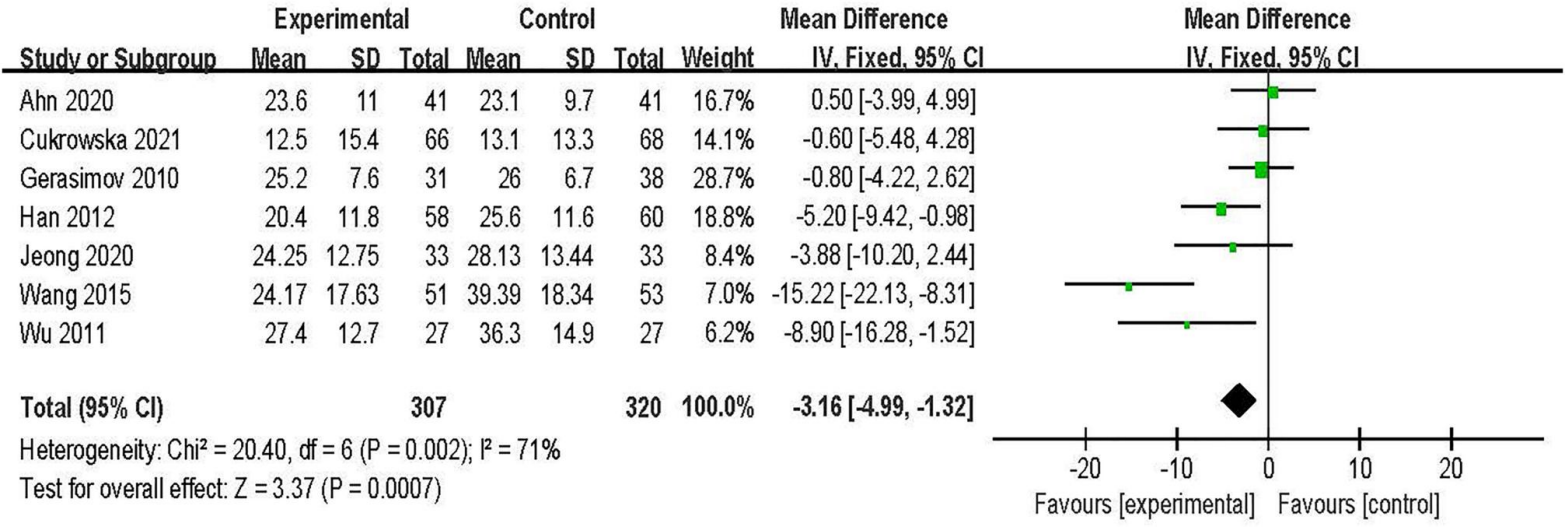
Forest plot showing the overall analysis of the SCORAD value.

A total of 10 outcomes from 9 RCTs were included. SCORAD was used as the evaluation or outcome index for the included studies. In total, 1000 patients were incorporated into the 9 RCTs. Three studies^26^, ^29^, ^32^ involving 373 patients were analyzed for dichotomous variables. Seven studies^25^, ^26^, ^27^, ^28^, ^30^, ^31^, ^33^ involving 627 children were analyzed for continuous variables. The outcomes of a meta‐analysis of the random‐effect model of dichotomous variables showed no significant differences between the probiotic and control groups [OR = 1.75, 95% CI (0.70, 4.35), *p* = 0.23, *I* ^2^ = 68%] (Figure 2); The results of the random‐effect model meta‐analysis of continuous variables indicated significant differences between the probiotic and control groups [MD = −4.24, 95% CI (−7.78, −0.71), *p* = 0.0007, *I* ^2^ = 71%] (Figure 3).

#### 3.4.2 Clinical effect by probiotic strain number

**FIGURE 4:**
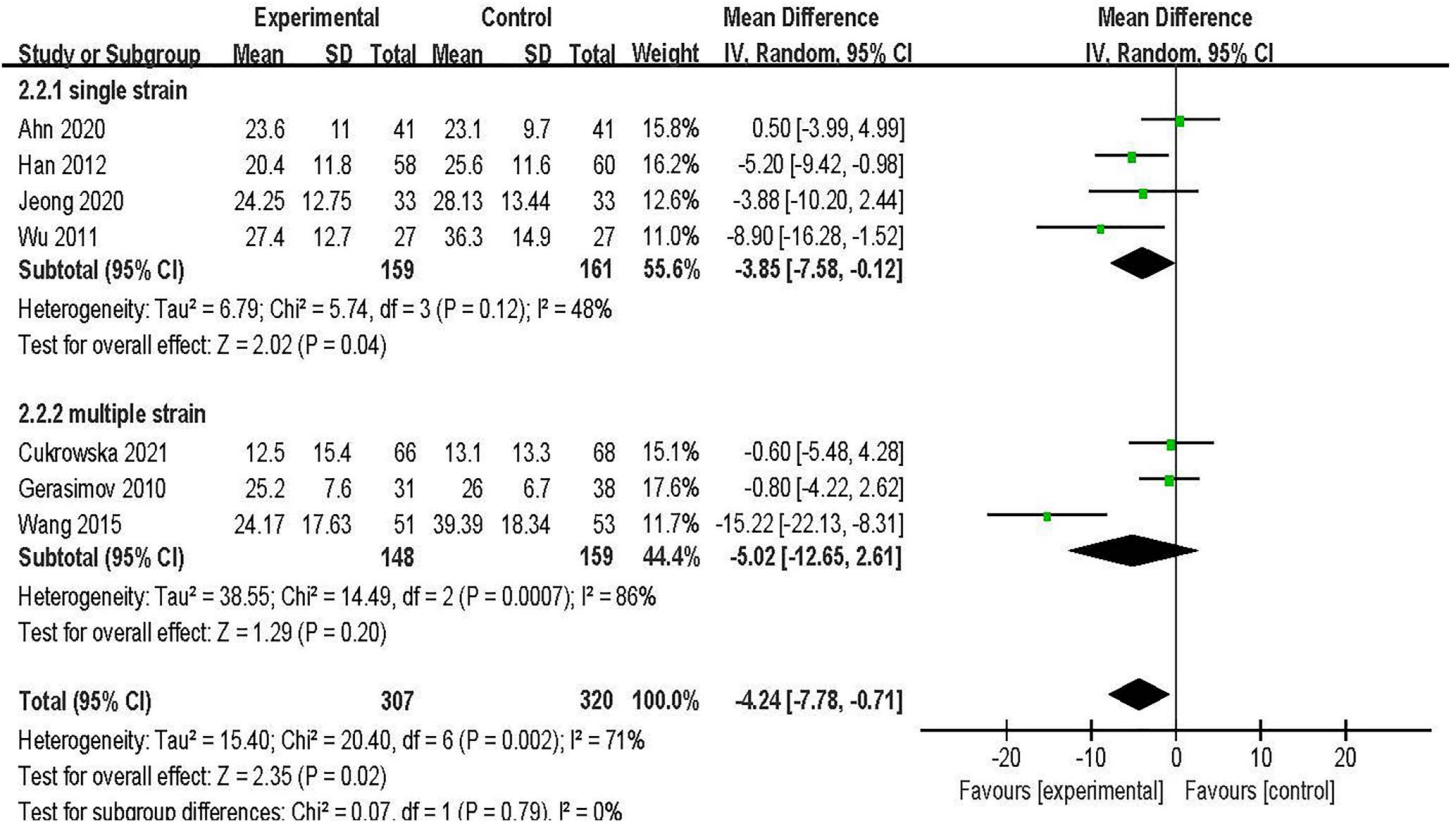
Forest plot showing subgroup analysis of SCORAD value by probiotic strain type.

Of the Seven studies, four^26^, ^27^, ^30^, ^31^ involving 320 patients used single‐strain probiotics, and three^25^, ^28^, ^33^ involving 307 patients used multi‐strain probiotics for AD. The difference between the use of single‐strain probiotics and the placebo group was statistically significant [MD = −3.85, 95% CI (−7.58, −0.12), *p* = 0.04, *I* ^2^ = 48%]. A subgroup analysis of probiotic types revealed no significant differences between the multi‐strains and the placebo groups [MD = −5.02, 95% CI (−12.65, 2.61), *p* = 0.2, *I* ^2^ = 86%]. Probiotics increased the SCORAD value, and there was a significant difference in the improvement of SCORAD between the single‐strain and the multi‐strain groups (*p* = 0.02) (Figure 4).

#### 3.4.3 Clinical effect by participant age

**FIGURE 5:**
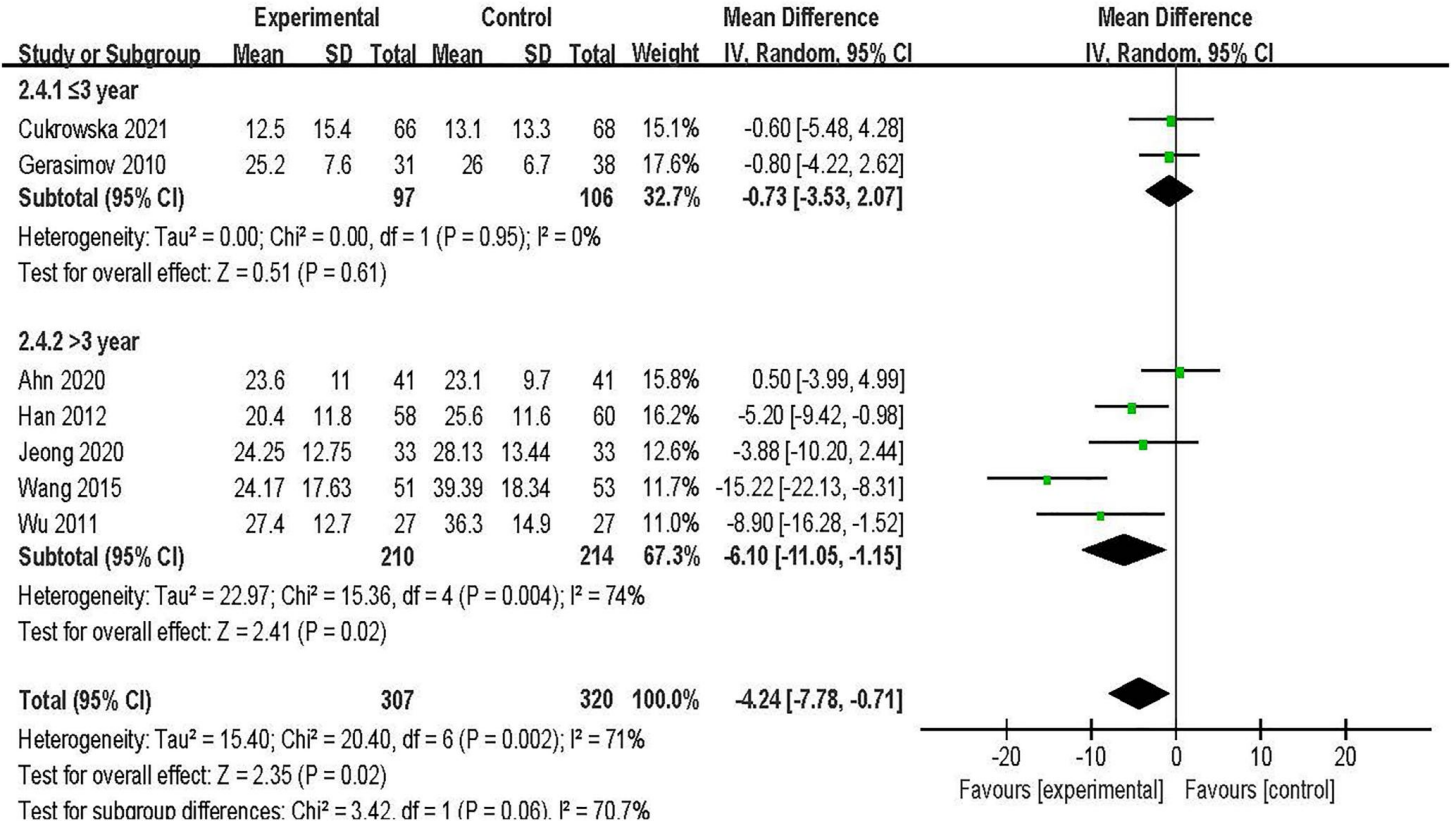
Forest plot showing subgroup analysis of SCORAD value by participant age.

Two studies^25^, ^33^ included children ≤3 years old, and another five studies^26^, ^27^, ^28^, ^30^, ^31^ included children >3 years old. The SCORAD values of the pediatric patients showed significant differences [MD = −6.10, 95% CI (−11.05, −1.15), *p* = 0.02, *I* ^2^ = 74%], whereas those of children ≤3 years old showed no significant differences [MD = −0.73, 95% CI (−3.53, 2.07), *p* = 0.61, *I* ^2^ = 0%]. Notably, the difference between subgroups was statistically significant (*p* = 0.02; Figure 5).

#### 3.4.4 Clinical effect by treatment duration

**FIGURE 6:**
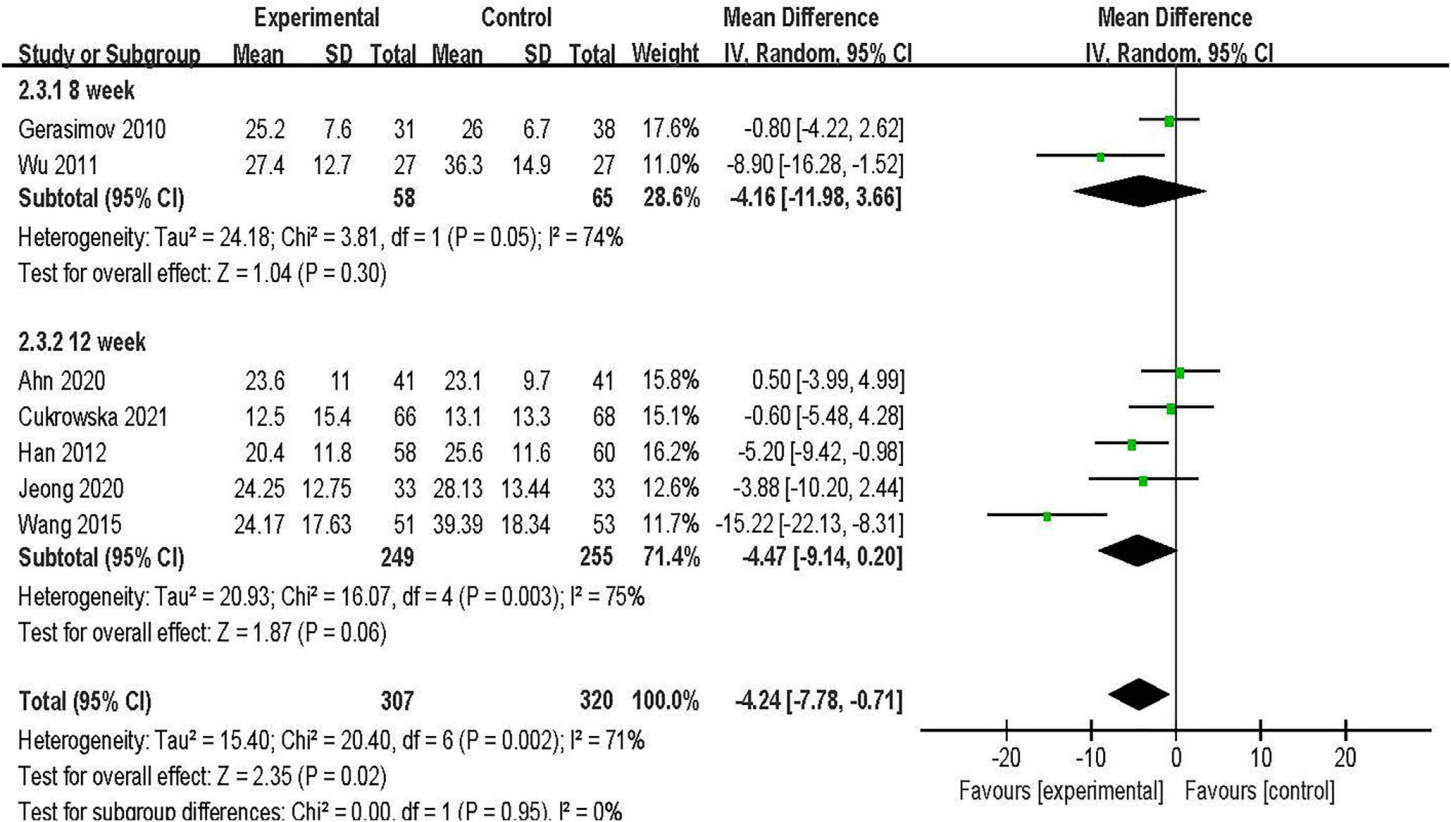
Forest plot showing subgroup analysis of SCORAD value by treatment duration.

In the meta‐analysis of seven continuous variables, two treatment durations emerged, namely 8 and 12 weeks, for which we performed subgroup analyses. Two studies had a treatment cycle of 8 weeks,^25^, ^27^ and 5 studies had a treatment duration of 12 weeks.^26^, ^28^, ^30^, ^31^, ^33^ No significant differences were found in the results of the treatment durations of 8 weeks [MD = −4.16, 95% CI (−11.98, 3.66), *p* = 0.3, *I* ^2^ = 74%] and 12 weeks [MD = −4.47, 95% CI (−9.14, 0.2), *p* = 0.06, *I* ^2^ = 75%]. The difference between subgroups was not statistically significant (*p* = 0.95; Figure 6).

### 3.5 Sensitivity analysis

Sensitivity analysis was performed by excluding a certain study to observe the heterogeneity of SCORAD values and analyze its main source. The sensitivity analysis of dichotomous variables was conducted. After excluding the study of Plummer,^32^ the difference between the two groups became statistically significant [OR = 2.90, 95% CI (1.35, 6.25), *p* = 0.006, *I* ^2^ = 0%]. This study enrolled preterm infants, which may have been a major source of heterogeneity in effects, considering the imperfect immune system function of this population. The sensitivity analysis of continuous variables was also performed. After removing the study of Wang,^28^ the heterogeneity was significantly reduced (*I* ^2^ = 36%). The SCORAD values in this study significantly differed between the probiotic and placebo groups. In studies that analyzed the effect of probiotics on AD severity, the SCORAD values were significantly lower in the pretreatment group than in the placebo group. However, at 3 months, the children in the probiotic group had lower SCORAD values than those in the placebo group. After 1 month of discontinuation of probiotics, no significant improvement in SCORAD value was observed in the probiotic group. After adjusting for age, sex and topical steroid use, the SCORAD scores remained significantly lower across groups. This result suggested that probiotics reduced serum Interleukin‐4 (IL‐4) and Tumor Necrosis Factor‐α (TNF‐α) but increased Interferon‐γ (IFN‐γ) and Transforming Growth Factor‐β levels at the end of treatment. Other serum indicators, except for IL‐4, were not statistically significant. Imbalances in gut microbiota composition and metabolic function due to dietary and lifestyle changes have been associated with the pathogenesis of atopic diseases. Activation of the IL‐4/IL‐13 axis in AD promotes the disruption of the skin barrier and is associated with changes in the gut microbiota.^34^, ^35^ The inconsistency of outcome measures may be the most important reason for heterogeneity. Another source of heterogeneity was that the patients did not completely suppress topical steroid use because of ethical concerns. We completed the meta‐analysis after removing this study with a greater effect on heterogeneity and found that the difference between the probiotic and control groups was still statistically significant [MD = −2.24, 95% CI (−4.15, −0.34), *p* = 0.002, *I* ^2^ = 36%].

## 4 DISCUSSION

AD is a common chronic inflammatory skin disease with heterogeneous clinical manifestations and multiple overlapping factors. It is associated with mutations in epidermal genes, skin barrier dysfunction, immune disorders, changes in lipid composition and gut microbial imbalances.^36^, ^37^, ^38^ The current understanding of AD etiology highlights gut microbial diversity, natural killer cell deficiency and immunophenotypes by age and ethnicity.^39^, ^40^, ^41^ A compromised skin barrier may be the first step in the development of AD, which leads to further skin inflammation and allergic sensitization. Type 2 cytokines, as well as IL‐17 and IL‐22, contribute to skin barrier dysfunction and AD development.^36^, ^42^ Evidence suggests that microbial exposure is important in AD development. Gut dysbiosis, which is a disequilibrium in gut microbial components and functions, has been implicated in the pathophysiology of various diseases, including AD and other allergic disorders in early childhood.^43^, ^44^, ^45^ The largest and earliest microbial exposure in humans is the gut microbiome.^12^ Gut microbiota is an important stimulator of the gut and immune development.^46^, ^47^ They continuously stimulate local and systemic immune responses, including the production of IgE and the formation of germinal centers within lymphoid follicles, through pattern recognition toll‐like receptors, thereby stimulating the development of gut‐associated lymphoid tissue.^48^ Ingestion of probiotics that help change the gut microbiota affects and interferes with AD.

With the progress of society and the development of civilization, people have become eager to treat diseases in a simple, cost‐effective and less harmful way. As early as 1997, a study published in the journal *Allergy Clin Immunol* suggested that probiotics promote endogenous barrier mechanisms and play a therapeutic role in patients with AD and food allergies.^49^ This study is the first to treat AD with probiotics. To clarify the mechanisms by which probiotics regulate the immune system, Viljanen et al. measured the expression levels of IL‐4, IL‐6 and TNF‐α and suggested that probiotics affect AD by relieving inflammation.^50^ The use of probiotics to treat or prevent allergic diseases has gradually become an option for people to fight allergic diseases, but some studies have challenged the role of probiotics in preventing allergies. Taylor et al. found that probiotic supplementation does not reduce AD risk in high‐risk infants and has been associated with increased allergen sensitivity in infants.^51^ Leontien et al. reviewed the clinical evidence for using probiotics to treat and prevent AD. Early studies showed insufficient evidence to support the treatment and prevention of AD with probiotics.^52^ To confirm the effect of probiotics on AD, related animal experiments were conducted. Kim et al. found that multi‐strain probiotics can regulate the balance of T cells in mice and improve the effect of AD.^53^ With the accumulation of evidence‐based data, Carol et al. analyzed the therapeutic effect of different strains of probiotics on AD and found that some strains exert a curative effect on AD.^54^ Most guidelines, including those from the European Society of Allergy and Clinical Immunology and the European Society of Paediatric Gastroenterology, Hepatology and Nutrition, do not recommend probiotic supplementation for the prevention of allergic disease because of the lack of clear evidence.^55^ In contrast, the World Allergy Organization recommends using probiotics for high‐risk infants because of its potential benefits for pregnant and breastfeeding women and infants to prevent AD. Given the possible synergies with other factors, careful selection of treatment strategies during pregnancy and early infancy is required to determine the optimal target population and ensure the effectiveness of probiotics for allergy prevention.^56^

This study summarizes the latest evidence on the clinical efficacy of probiotics in children with AD from 2010 to 2023. We performed meta‐analyses of dichotomous and continuous variables separately in the included studies. The clinical effect of probiotics in the treatment of AD in children with dichotomous variables was not statistically significant, and the meta‐analysis of continuous variables showed that probiotics were significantly effective in treating AD in children. Considering the high heterogeneity of the results for continuous variables, we performed subgroup analyses based on the number of strains, the age of the children and the duration of treatment. Several issues were identified in the subgroup analyses. First, we found a statistically significant improvement in the SCORAD value with single‐strain probiotics. Doege^57^ et al. performed meta‐analyses of the effect of probiotic types on AD; the results showed that supplementation with probiotics significantly reduces the risk of AD. However, this effect was only significant for single‐strain probiotics and not for multi‐strain probiotics. Conversely, the current evidence does not show that the prognosis of children with AD is related to treatment duration and age. Michail et al.^58^ conducted a meta‐analysis and showed no significant differences in the SCORAD values of children treated for less than and more than 6 weeks. No significant correlation was found between age and SCORAD values. Kim et al.^59^ performed a subgroup analysis and showed that the improvement in AD is significant when the treatment duration is more than 8 weeks, but not significant when the treatment duration is less than 8 weeks. The heterogeneity of RCTs related to treatment time is not significant, and treatment cycles longer than 8 weeks may have a better effect than treatment cycles shorter than 8 weeks. Zhao et al.^60^ found a significant reduction in SCORAD value in infants with treatment cycles ≤8 weeks. The results of our meta‐analysis are similar to those of Michail's, except for some differences in the definition of treatment cycles. In addition, the meta‐analysis of Michail et al. included small‐scale studies with high heterogeneity, and the reliability of the results warrants large‐scale prospective studies for further confirmation. However, the study of Zhao was limited to infants, and the analysis results could not provide a comprehensive and accurate assessment of the SCORAD value in childhood.

Zhao et al.^60^ reported in their meta‐analysis that probiotic therapy may be more effective in children aged 1 year or less. In a meta‐analysis with a large number of included studies, probiotics significantly improved the SCORAD value in children over 1 year of age but not in infants under 1 year of age. Notably, the subgroup differences were significant.^61^ This study supports the findings of our meta‐analysis. Huang et al. also reported no significant effect of probiotics on AD children (<1 year).^62^ Our results are similar to Huang and Zhao's findings; probiotics only reduced the SCORAD value in children aged >3 years, but the difference was not statistically significant. Based on the assumption that probiotics are effective during early development, the establishment and composition of the gut microbiota are typically completed at 2–3 years of age, and the immune system is programmed for future use at this stage. After the age of 3 years, the gut microbiota is completely established, which contributes to the exertion of probiotics.^63^ Not all studies support our findings. Studies have shown that strains in children's guts change dramatically during the first few weeks of life and after weaning. Probiotics can influence and alter the gut microbiome of children more than adults.^64^, ^65^ However, this finding does not mean that the younger the patients, the better the improvement of the SCORAD value. Further clinical studies are necessary to confirm this result.

Due to the diversity of probiotic species, multiple probiotic species emerged at the time of the intervention. Among these species, *Lactobacillus acidophilus* and *Bifidobacterium lactis* have the advantage of improving intestinal diseases.^66^ *L. acidophilus* DDS‐1 can induce gut microbial ecological interactions in mice.^67^ *L. rhamnosus* is a widely used probiotic. Its effector molecule is lipoteichoic acid, which stimulates a strong immune response. Although its immune effect is controversial, it is still used in therapy or the prevention of certain allergic diseases.^68^ *L. rhamnosus* also exerts a therapeutic effect on AD.^31^ A report in 2019 found that *Lactobacillus fermentum* can regulate immune metabolism and reduce the expression of inflammatory factors.^69^ Considering the lack of global consensus on the names of strains and the wide variety of strains, this study could not evaluate the therapeutic effect of specific strains on children with AD, which is also the main limitation of this study.

This study has some limitations. First, the total number of included studies and the sample scales were relatively small, which affected the reliability of the conclusions. Second, some of the included studies did not report allocation concealment, which has a certain risk of bias. Third, the number, types and administration methods of probiotics in different studies may also lead to clinical heterogeneity.

## 5 CONCLUSION

This study showed that single‐strain probiotic treatment exerts a positive effect on the AD. Few studies focused on the improvement of probiotics with AD in children, and future investigators should adopt large sample sizes and extend follow‐up time to assess the mechanisms of action and long‐term effects of probiotics. Research on AD prevention in children should also be increased to explore data for improving children's health. Defined by the quantity and quality of the included studies, the conclusions of the present work warrant further verification by high‐quality studies.

## AUTHOR CONTRIBUTIONS

Xiali Xue and Xinwei Yang designed this study. Xiali Xue finished the manuscript. Xiali Xue, Xinwei Yang, and Zhongyi Deng revised the manuscript. Xiubo Shi, Xinwei Yang, Xiali Xue, and Zhongyi Deng collected and analyzed the data. All authors critically commented on drafts of the manuscript.

## CONFLICT OF INTEREST STATEMENT

None of the authors have any competing interests to declare.

## Supporting information

Supplementary Material S1

Supplementary Material S2

## Data Availability

Data sharing is not applicable to this article as no datasets were generated or analyzed during the current study.
