# Supplementary material for: Efficacy of probiotics in pediatric atopic dermatitis: A systematic review and meta‐analysis
Source: Clin Transl Allergy. 2023 Jul 15;13(7):e12283. doi: 10.1002/clt2.12283 (PMC10349543; doi:10.1002/clt2.12283)
Supplement: Supplementary file 2 — Supplementary Material S2 [file CLT2-13-e12283-s002.docx]

Search strategy for the PubMed database.

("Probiotic"[Title/Abstract] OR "Probiotics"[Title/Abstract]) AND ("dermatitis atopic"[Title/Abstract] OR (("Atopic"[All Fields] OR "atopical"[All Fields] OR "atopics"[All Fields]) AND "Dermatitides"[Title/Abstract]) OR "atopic dermatitis"[Title/Abstract] OR (("dermatiti"[All Fields] OR "Dermatitis"[MeSH Terms] OR "Dermatitis"[All Fields] OR "Dermatitides"[All Fields]) AND "Atopic"[Title/Abstract]) OR "neurodermatitis atopic"[Title/Abstract] OR "atopic neurodermatitis"[Title/Abstract] OR (("Neurodermatitis"[MeSH Terms] OR "Neurodermatitis"[All Fields]) AND "Atopic"[Title/Abstract]) OR "neurodermatitis disseminated"[Title/Abstract] OR "disseminated neurodermatitis"[Title/Abstract] OR (("Neurodermatitis"[MeSH Terms] OR "Neurodermatitis"[All Fields]) AND "Disseminated"[Title/Abstract]) OR "eczema atopic"[Title/Abstract] OR "atopic eczema"[Title/Abstract] OR "eczema infantile"[Title/Abstract] OR "infantile eczema"[Title/Abstract] AND "Randomized controlled trial "[Title/Abstract]) OR "Randomized"[Title/Abstract] OR " Randomly"[Title/Abstract] OR "Clinical trial"[Title/Abstract]).
